# Aryl Hydrocarbon Receptor (AHR) is required for repopulation of decellularized intestinal colon scaffolds

**DOI:** 10.17912/micropub.biology.001529

**Published:** 2025-04-25

**Authors:** Lizbeth Perez-Castro, Busola Alabi, Afshan Nawas, M.Carmen Lafita-Navarro, Jerry Shay, Maralice Conacci-Sorrell

**Affiliations:** 1 The University of Texas Southwestern Medical Center, Dallas, Texas, United States

## Abstract

This study investigates the role of the ligand-activated transcription factor AHR in repopulating the intestinal lining. Using organoid-derived cells and decellularized mouse intestinal scaffolds to investigate the importance of AHR in regulating intestinal regeneration, we found that silencing AHR expression hinders the capacity of colonic cells to repopulate decellularized colons. We therefore propose that AHR may play an important role in regulating intestinal regeneration. The ligand-dependent nature of AHR activity may provide an opportunity to interfere with disorders such as cancer and inflammatory bowel diseases which are caused by dysregulation in intestinal tissue renewal.

**Figure 1. AHR downregulation prevents repopulation of intestinal scaffolds by cultured colonic cells:**
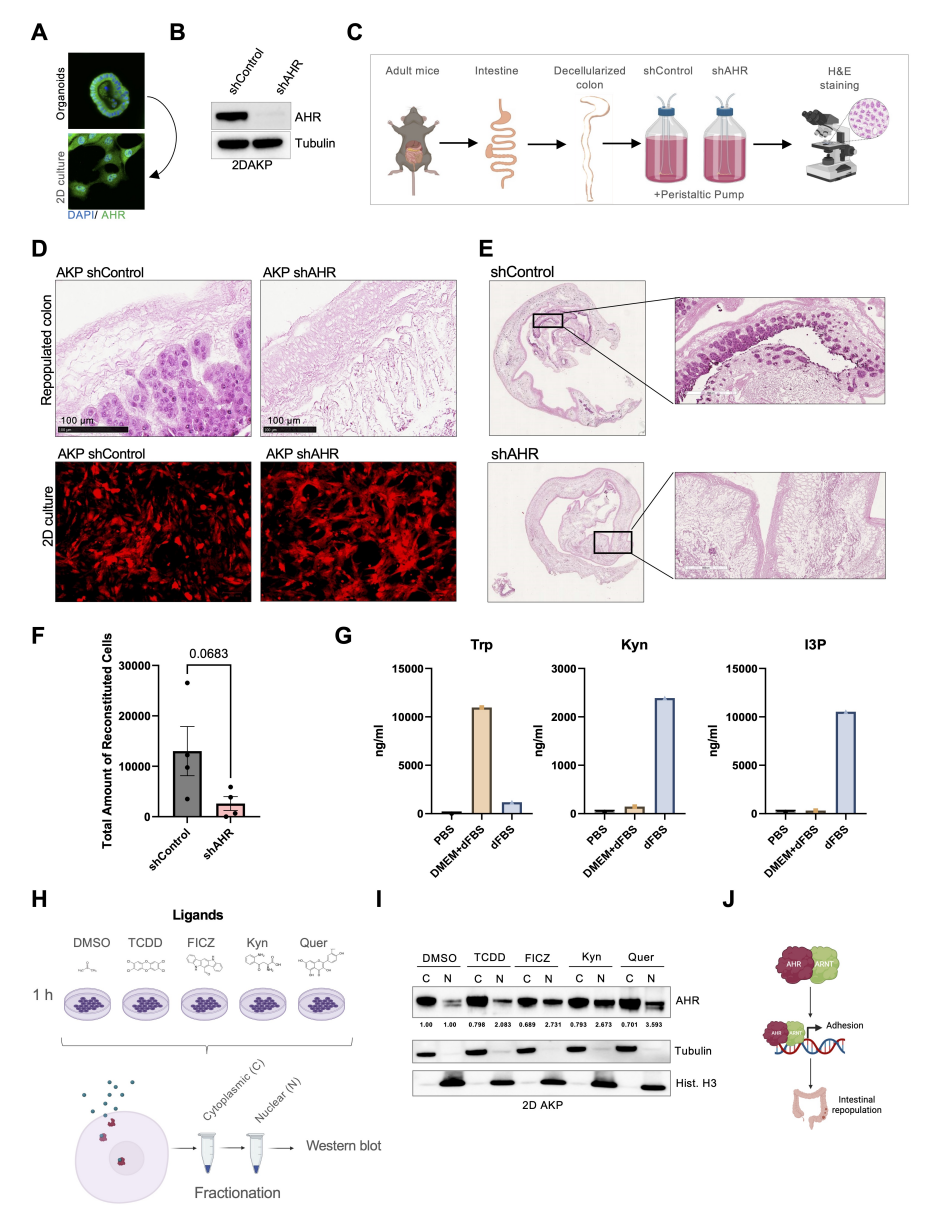
A) Immunofluorescence images of AKP in 3D and 2D culture. Immunofluorescence for AHR is shown in green and DAPI in blue. B) Western blot of 2D AKP lysates of cells infected with lentiviral particles for control or AHR shRNA. C) Schematics for decellularization experiments. Mouse colons were decellularized and used for repopulation studies for three days, followed by fixation and H&E staining to evaluate cellular infiltration and tissue architecture. D) H&E and 2D culture staining of the repopulation experiments done using 2D AKP cells infected with a control or AHR shRNA. Bottom panel shows the culture growing in 2D expressing TdTomato. E) H&E of the entire slide of repopulation experiment performed with 6 million AKP cells for 3 days. F) Quantification of cells that repopulated the intestinal lining (N=3 independent experiments performed). G) Tandem mass spectrometry, LC-MS/MS used to quantify Trp metabolites in PBS (negative control) DMEM supplemented with 5% FBS and undiluted FBS. H) Schematic of cellular fractionation after AHR activation through ligand exposure. I) Western blot for AHR in nuclear (N) and cytoplasmic (C) fractions lysates extracted from cells incubated for 1 hour with AHR ligands. N=3 independent experiments performed. Nuclear fractions were quantified and normalized by histone H3signal and cytoplasmic fractions were quantified and normalized by tubulin signal. J) Working model for intestinal repopulation showing AHR activation by ligands drive its nuclear translocation and promotes engraftment of 2D AKP cells.

## Description

The intestine sustains continuous renewal of its lining through tightly regulated cell proliferation and differentiation (Barker et al., 2007; Shaker & Rubin, 2010; van der Flier et al., 2009). Misregulation of these processes can lead to cancer and inflammatory bowel diseases, including colitis and necrotizing enterocolitis (Hong et al., 2017). Maintaining a healthy intestinal lining through rapid turnover is important to facilitate and increase the absorption of nutrients. Small metabolites derived from dietary components and the gut microbiome contribute to the maintenance of the intestinal lining (Alvarado et al., 2019; Metidji et al., 2018). Some of these metabolites, including tryptophan-derived products found in the intestinal environment, such as kynurenine (Kyn), indole 3 pyruvate (I3P), and 6-Formylindolo[3,2-b]carbazole (FICZ), function as ligands for AHR promoting its nuclear translocation and the transcriptional activation of its target genes (Murray et al., 2014; Dai et al., 2022; Rannug et al. 2018 and 1987). Previous work in our lab revealed that AHR and its tryptophan-derived ligand, Kyn, play an important role in colonic cell growth, and thus AHR acts as a central integrator of growth signaling in colon cancer (Lafita-Navarro et al., 2018; Perez-Castro et al., 2021; Venkateswaran et al., 2019b). However, the specific molecular mechanism by which AHR affects the behavior of normal stem and progenitor cells is not fully understood.

Using primary organoids, we attempted to repopulate decellularized colonic scaffolds. However, low proliferation rates and limited cell yields hindered our ability to generate enough cells for successful repopulation. We also attempted to repopulate colonic mouse scaffolds using AKP [(APC−/− (CRISPR), p53−/− (floxed allele, excised), KRas ^G12D^ (activated from LSL-KRasG12D), and TdTomato red (From Rosa-LSL-TdTomato)] colonic organoids, which are partially transformed, and thus grow faster. However, cell numbers were still insufficient for robust repopulation. Therefore, we established a 2D culture of these organoids by adapting them to grow attached to plastic tissue culture plates (hereafter referred as 2D AKP cells). These cells were more amenable for large scale culturing, allowing us to obtain enough cells for repopulation studies ( Fig. 1 A). We generated stable 2D AKP cells expressing control shRNAs or an shRNA targeted to knockdown AHR ( Fig. 1 B). These cells were used to repopulate colonic tissues.

Using a previously described system (Alabi et al., 2019) we decellularized mice colon leaving the ECM and architecture of the intestinal lining intact. For repopulation studies, we flushed 2D AKP cells expressing control shRNA or AHR shRNA through the decellularized colons for 3 days using a peristaltic pump ( Fig. 1 C). This experiment revealed the ability of 2D AKP cells to repopulate the decellularized colons ( Fig. 1 D-F) measured by the presence of cells stained with hematoxylin and eosin (H&E) within the colon scaffold. For this quantification, slides of H&E-stained colonic tissues were scanned and cells were enumerated comparing control and shRNA samples ( Fig. 1 F). Importantly, although AHR knockdown cells were unable to repopulate decellularized colonic scaffolds ( Fig. 1D, top images), they grew just as well as control cells when cultured on 2D tissue culture plates ( Fig. 1D, bottom images), thus indicating an important role for AHR for the growth of colonic cells in 3D matrices.

Interestingly, we found that the DMEM culture media used for the long-term repopulation cultures contains endogenous ligands of AHR including I3P and Kyn (Sadik et al., 2020) ( Fig. 1 G) which, we surmise, have the ability to activate AHR in control 2D AKP cells during these experiments. To ensure that these 2D AKP cells have functional AHR molecules, we incubated these cells with small molecules known to promote AHR nuclear translocation, including TCDD, FICZ, Kyn, and quercetin ( Fig. 1 H) and following 1 hour incubation, nuclear and cytoplasmic fractions were isolated, and lysates were analyzed by Western blot. These results confirmed that AHR nuclear translocation is enhanced upon addition of ligands to the culture media ( Fig. 1 I). These results indicate that AHR is activatable in the 2D AKP cells and likely respond to serum-derived small molecule ligands to promote a signaling cascade that drives the repopulation of the colonic scaffold ( Fig. 1J, proposed model).

Our results enforce the notion that AHR regulates intestinal homeostasis likely through controlling the transcription of target genes (Wiggins et al., 2023), thus future studies targeted at understanding the specific pathways regulated by AHR in concert with its ligands may lead to new therapies to treat intestinal diseases like inflammatory bowel diseases. Moreover, our studies may serve as the foundation for future ex-vivo studies targeted at improving the ability of patient-derived cells to regenerate damaged intestinal tissue.

## Methods

Colonic repopulation

Colonic tissues were harvested from C57 black 6 mice under UTSW IACUC approved protocol number 2016-101375 and used for colonic decellularization following a previously established protocol (Alabi et al., 2019) using a peristaltic pump (Cole Parmer: Masterflex EW-07522-30), an autoclavable glass jar, and a rubber stop-cork housing silicone tubings (Cole Parmer: Masterflex EW-96410-14). The tubings connected to the peristaltic pump serves to transport decellularization agents in a circular motion from the glass jar, through the colon, and back into the jar. First, isolated colons were flushed with 1X PBS + 5% antibiotics and antimycotics (5XPBSAA) (Gemini Bio Products, CA) using a 30 ml syringe (BD: 309650) to remove bacteria and fecal matter, repeated up to three times. Then colons were connected to the peristaltic pump via the decellularization tubing in the bioreactor jar and distilled water was used for perfusion overnight at 4°C, followed by perfusion with 4% sodium deoxycholate for 2 hours at room temperature and then by 1% antibiotic and antimycotic solution for 30 minutes at RT. Then the tissues were further perfused with 2000 units of DNAse for 1.5 hours. Colons were stored overnight at 4°C in a bioreactor jar containing antibiotics and antimycotic solution. This protocol ensures full removal of all colonic cells with only scaffolds remaining (Alabi et al., 2019), thus allowing the repopulation of the tissues only by the exogenously provided cells. Decellularized colons were conditioned in Dulbecco's modified Eagle's medium (DMEM) for 1 hour in a 37°C and 5% CO2 incubator and then coated with fibronectin (Thermofisher Scientific, MA, 33016015). For the repopulation experiments, the bioreactor jar was filled with ~150 ml of DMEM medium and colons were connected to the perfusion pump. 1.2 ml of medium with single cell suspensions (6 million 2D AKP cells shControl and shAHR) were pumped through the lumen of decellularized colons at a flow rate of 0.04 ml/min for 2 hours for 3 days.

AKP organoid culture

Mouse organoids generated from the following backgrounds: APC−/− (CRISPR), p53−/− (floxed allele, excised), KRas ^G12D^ (activated from LSL-KRasG12D), and TdTomato red (From Rosa-LSL-TdTomato) (Venkateswaran et al., 2019a). AKP organoids were maintained at 37°C as 3D spheroid culture in Matrigel. These were cultured in advanced DMEM/Ham’s F-12 supplemented with 1× penicillin/streptomycin and 1×/2 mM glutamax. Minimal basal medium supplemented with 1× B27 and 10 µM Y-27632 was used for Matrigel drops. For 2D growth of AKP organoids, single cells suspensions were seeded in six-well cell culture plates at 50,000 cells per well. Established cell cultures were maintained in DMEM high glucose supplemented with penicillin/streptomycin and 10% fetal bovine serum (FBS).

For AHR knockdown, recombinant lentiviruses were produced by transfecting HEK293T Phoenix-amphotropic packaging cells with pMD2G (VSV-G protein, Addgene, #12259), pPAX2 (lentivirus packaging vector, Addgene, #12260), and lentiviral constructs using Lipofectamine 3000 (Thermo Fisher Scientific, L3000015). Media containing lentiviruses were harvested 48 h and 72 h after transfection, filtered, and used to infect 2D AKP cells, which were then selected with puromycin to establish stable cell lines. Control shRNA lentiviral vector was purchased from Sigma-Aldrich (SHC216; MISSION® TRC2 pLKO.5-puro non-target shRNA Control Plasmid DNA). AHR shRNA (GTCAAGCCTGTTAGCTATATT) lentiviral vector was purchased from Sigma-Aldrich (TRCN0000218025). For viral transduction, 50,000 cells were mixed with 10 μg/mL Polybrene and pLKO or shRNA-containing virus. Infected 2D AKP cells were selected with puromycin and maintained in DMEM with 10% FBS and 100 units/mL penicillin/streptomycin.

Metabolite LC/MS

For quantification of Trp metabolites present in DMEM containing 10% FBS, metabolites were extracted from 100 µL of media with 400 µL of methanol. The supernatant was dried down by speedvac and the dried extraction supernatant was resuspended in 200 µL ddH2O + 25 ng/mL tolbutamide IS + 10 ng/mL d5 Trp IS + 100 ng/mL d5 IAA IS. The material was dissolved by vortexing and room temperature incubation for 10 min at 37. The samples were spun at 14,000 rpm for five minutes and the supernatant was kept. Kyn, I3P, and Trp were measured using HPLC plate and analyzed by LCMS as previously described (Mullen et al., 2014).

Western blotting

For total cell lysates, cells were lysed using RIPA buffer (25 mM Tris-HCl pH 7.4, 150 mM NaCl, 1% NP-40, 0.5% sodium deoxycholate, 0.1% SDS + protease and phosphatase inhibitors and MG132). For nuclear and cytoplasmic fractionations, cytoplasmic extracts were collected using buffer A (10 mM HEPES, 10 mM KCl, 1.5 mM MgCl _2_ , 0.5% [10% NP-40]). The buffer was added to the cells, incubated on ice for 20 minutes, and then centrifuged at 4°C for 4 minutes at 4000 rpm to obtain the supernatant or cytoplasmic fraction. The residual pellets were then washed twice with buffer A, resuspended in RIPA, sonicated, and centrifuged at 21,130 g for 15 minutes to collect nuclear fractions from the supernatants. Both buffers were supplemented with protease and phosphatase inhibitors. For Western blotting, samples were run on a 4-12% gradient acrylamide gel and transferred to a nitrocellulose membrane. Membranes were blocked with 10% milk or 5% BSA in Tris-buffered saline containing 0.05% Tween 20 (TBS-T) for 1 hour at room temperature or overnight and incubated overnight with primary antibodies dissolved in TBS-T containing 1% BSA. Membranes were then washed three times with TBS-T for 10 minutes and incubated for 1 hour at room temperature with secondary antibodies dissolved in 5% milk or 1% BSA in TBS-T. Membranes were again washed three times with TBS-T for 10 minutes and visualized using the Bio Rad Chemidoc system. The following primary antibodies were used: anti-AHR polyclonal antibody (1:1000 Enzo Life Sciences 50-201-1848), anti-histone H3 antibody (1:2000 Cell Signaling, 4499S), and anti-tubulin antibody (1:5000 Sigma T6199-200ml). The following secondary antibodies were used: for western blotting mouse secondary m-IgGk BP-HRP (Santa Cruz Biotechnology, sc-516202), sheep anti-mouse IgG1-HRP (Abcam, ab6808), donkey anti-rabbit IgG HRP (Abcam, ab16284). The abundance of AHR in nuclear and cytoplasmic was calculated using ImageJ. Signals were normalized by loading controls; cytoplasmic fractions were normalized by tubulin and the nuclear fractions by histone H3 levels. The levels of AHR in DMSO control samples were set to 1 and AHR levels in samples incubated with AHR ligands are presented as a function of DMSO. All experiments were performed three times.

**Immunofluorescence and H&E staining**

Cells were fixed with 4%paraformaldehyde, permeabilized with 0.5% Triton X-100, and blocked with 5% BSA for 1h at room temperature. Samples were incubated with primary antibody for 1 hour at room temperature or overnight at 4°C. Primary antibody used was anti-AHR polyclonal antibody (1:1000 Enzo Life Sciences 50-201-1848). Alexa Fluor 488 (Fisher, A11008) secondary (1:500) was used (incubated for 1 hour) and DAPI (1 µg/ml) was added immediately before imaging. Images were taken with an inverted confocal microscope. For H&E staining, repopulated colons were fixed in formalin for at least 24 hours and processed into paraffin blocks and processed tissue were sectioned into 5 µm sections that were heat fixed, rehydrated and H&E staining were performed following established protocols (Venkateswaran et al., 2024).
